# The regulation of connective tissue growth factor expression influences the viability of human trabecular meshwork cells

**DOI:** 10.1111/jcmm.12492

**Published:** 2015-02-20

**Authors:** Sabrina Kuespert, Benjamin Junglas, Barbara M Braunger, Ernst R Tamm, Rudolf Fuchshofer

**Affiliations:** Institute of Human Anatomy and Embryology, University of RegensburgRegensburg, Germany

**Keywords:** CTGF, glaucoma, trabecular meshwork, endothelin-1, angiotensin-II, IGF-1, oxidative stress, heat shock, αB-crystallin, cell viability

## Abstract

Connective tissue growth factor (CTGF) induces extracellular matrix (ECM) synthesis and contractility in human trabecular meshwork (HTM) cells. Both processes are involved in the pathogenesis of primary open-angle glaucoma. To date, little is known about regulation and function of CTGF expression in the trabecular meshwork (TM). Therefore, we analysed the effects of different aqueous humour proteins and stressors on CTGF expression in HTM cells. HTM cells from three different donors were treated with endothelin-1, insulin-like growth factor (IGF)-1, angiotensin-II, H_2_O_2_ and heat shock and were analysed by immunohistochemistry, real-time RT-PCR and Western blotting. Viability after H_2_O_2_ treatment was measured in CTGF silenced HTM-N cells and their controls. Latrunculin A reduced expression of CTGF by about 50% compared to untreated HTM cells, whereas endothelin-1, IGF-1, angiotensin-II, heat shock and oxidative stress led to a significant increase. Silencing of CTGF resulted in a delayed expression of αB-crystallin and in reduced cell viability in comparison to the controls after oxidative stress. Conversely, CTGF treatment led to a higher cell viability rate after H_2_O_2_ treatment. CTGF expression is induced by factors that have been linked to glaucoma. An increased level of CTGF appears to protect TM cells against damage induced by stress. The beneficial effect of CTGF for viability of TM cells is likely associated with the effects on increased ECM synthesis and higher contractility of the TM, thereby contributing to reduced aqueous humour outflow facility causing increased intraocular pressure.

## Introduction

Primary open-angle glaucoma (POAG), one of the major causes of blindness worldwide 1, is a neuropathy of the optic nerve leading to a loss of axons at the optic nerve head. The critical risk factor for POAG is intraocular pressure (IOP) which is frequently elevated 2–4. Elevated IOP is caused by an abnormally high aqueous humour (AH) outflow resistance that is generated in the juxtacanalicular region of the human trabecular meshwork (HTM) 5,6. To date, the mechanisms that are responsible for the increase in TM outflow resistance in POAG are not fully understood 7,8. There is some evidence though that changes in the amounts and the composition of the HTM extracellular matrix (ECM) 9,10 as well as in the actomyosin system of HTM cells 11–14 are involved.

Currently, transforming growth factor (TGF)-β2 is one of the leading candidates among the multiple signalling molecules in the AH that may cause molecular changes leading to an increase in outflow resistance in POAG. Accordingly, patients suffering from POAG exhibit higher levels of TGF-β2 in the AH when compared to healthy controls 15–18. TGF-β2 is a strong inducer of the HTM ECM and a modifier of the actin cytoskeleton in TM cells 19. Perfusion of anterior segments with TGF-β2 results in an increase in outflow resistance 20.

In recent studies, we showed that connective tissue growth factor (CTGF) is mediating most of the ECM effects of TGF-β2 on TM cells 21. In addition, CTGF is able to modulate the biological properties of the TM actin cytoskeleton and to increase its contractility 11. CTGF belongs to a family of regulatory proteins that are upregulated in a substantial number of disorders associated with a pathological increase in ECM 22–24. In the HTM, CTGF is among the most highly expressed genes 25. The comparison of the CTGF expression in Schlemm's canal endothelial (SC) cells derived from glaucoma patients with SC cells from healthy donors reported a significant higher CTGF expression level in the glaucomatous SC cells 26. CTGF has also been detected in the AH 27 and its amounts are increased in patients with pseudoexfoliation syndrome and glaucoma associated with it 28,29. In patients with POAG, only a slight increase in CTGF was found in the AH in one study 28, whereas other authors reported a significant increased level of CTGF 30.

To investigate the influence of CTGF signalling in the living eye, we recently developed a mouse model with an eye-specific overexpression of CTGF by the use of a lens-specific promotor. Since the CTGF-mediated effects on ECM and the actin cytoskeleton that correlated with an increase in IOP and a successive loss of axons in the optic nerve, we concluded that high amounts of CTGF cause POAG in the mouse eye 11.

The dynamics of factors that may increase the trabecular expression of CTGF in patients with POAG remain unclear. TGF-β and cyclic mechanical stress are the only known stimuli that have been shown to increase the expression of CTGF in HTM cells 31,32. In the present study, we show that the regulation of CTGF expression involves various factors including different kinds of stress, suggesting a protective role for CTGF in the HTM.

## Material and methods

### Cell culture

Cultures of HTM cells were established from the eyes of three human donors according to protocols published previously 32. The age of the donors ranged from 34 to 76 years. HTM cells of the third to fifth passage were seeded in 35-mm culture wells (4.0 × 10^5^ cells/well) and grown to a confluent monolayer in F10-HAM medium plus 10% (v/v) foetal bovine serum without antibiotics in 5% CO_2_ at 37°C (PAA, Pasching, Austria). The confluent cells were incubated in serum-free medium for 24 hrs followed by incubation in fresh serum-free medium. Endothelin-1 (ET-1), angiotensin-II (Ang II), insulin-like growth factor (IGF)-1, hydrogen peroxide (H_2_O_2_) or latrunculin A (LatA; Sigma-Aldrich, Taufkirchen, Germany) were added at various concentrations at different time-points. Control cells were treated equally with corresponding vehicles.

Heat-shock experiments were performed by seeding immortalized HTM cells (HTM-N) 33 and pSiCTGF-HTM-N cells (stable plasmid-based siRNA silencing of the CTGF gene) into Petri dishes to a confluent level (1.0 × 10^6^ cells/well). The pSiCTGF-HTM-N cell line has an 80% reduced CTGF expression in comparison to the HTM-N cell line as described previously 11. The cells were kept for 24 hrs under serum-free conditions. Then, cells were heat shocked for 15 min. at 42°C in a water bath and harvested after a further 37°C incubation period at different time-points [0 (control), 15, 30, 45, 60 min.].

Each of the described experiments except heat shock was done with each of the three primary cell lines. Methods for securing human tissues were humane, included proper consent and approval, and complied with the Declaration of Helsinki.

### RNA analysis

Human trabecular meshwork cells were harvested and total RNA was extracted with TRIzol (Invitrogen, Karlsruhe, Germany) according to manufacturer's recommendations. First strand cDNA was prepared from total RNA using the iScript cDNA Synthesis Kit (Bio-Rad, Munich, Germany) according to the manufacturer's instructions. Real-time RT-PCR was performed on a BioRad iQ5 Real-time PCR Detection System (Bio-Rad) using the following temperature profile: 40 cycles of 10 sec. melting at 95°C, 40 sec. of annealing and extension at 60°C. Primer pairs (Table1) were purchased from Invitrogen and extended over exon–intron boundaries. RNA that was not reversely transcribed served as negative control for real-time RT-PCR. Glyceraldehyde-3-phosphate dehydrogenase (GAPDH) and guanine nucleotide binding protein 2-like-1 (GNB2L1) were both used as housekeeping genes for relative quantification of the real-time RT-PCR experiments. Quantification was performed with iQ5 Standard-Edition (Version 2.0.148.60623) software (Bio-Rad).

**Table 1:** Sequences of primers used for real-time RT-PCR

Type	Sequence	Position	T_m_ (°C)
CTGF	5′-CTCCTGCAGGCTAGAGAAGC-3′	884–977	59
	5′-GATGCACTTTTTGCCCTTCTT-3′		60
GAPDH	5′-AGCCACATCGCTCAGACA-3′	83–148	60
	5′-GCCCAATACGACCAAATCC-3′		60
GNB2L	5′-GCTACTACCCCGCAGTTCC-3′	170–241	59
	5′-CAGTTTCCACATGATGATGGTC-3′		60

### Western blot analysis

To obtain protein extracts, cells were directly lysed in RIPA lysis buffer (150 mM NaCl, 1% NP-40, 0.5% deoxycholic acid, 0.1% SDS and 50 mM Tris, pH 8) and protein content was measured with the bicinchoninic acid protein assay (Pierce, Rockford, IL, USA). Proteins were separated by SDS-PAGE and transferred to polyvinylidene fluoride membranes. Antibodies were used as follows: rabbit anti-human αB-crystallin (1:200; Stressgen, San Diego, CA, USA), goat anti-human CTGF (1:500), donkey anti-rabbit-horseradish peroxidase (HRP) and chicken anti-goat-HRP (1:2000; all Santa Cruz, CA, USA). Chemiluminescence was detected on a LAS 3000 imaging workstation (Raytest, Straubenhardt, Germany). For normalization of the signals, blotted membranes were stained with coomassie blue and digitized. The total amount of protein per lane was determined and calculated using AIDA Image analyser software (Raytest). The values of the total amount of protein were used to normalize the signal intensity of the bands detected in Western blot analysis.

### Immunohistochemistry

Cultured HTM-N and pSiCTGF-HTM-N cells were grown on microscope slides and treated with heat shock as described above. After incubation, cells were fixed with 4% paraformaldehyde for 15 min. and subsequently washed twice with PBS containing 0.1% Triton X-100. Rabbit anti-human αB-crystallin antibodies were added at a 1:50 dilution in PBS/bovine serum albumin (BSA; 5%) for 4 hrs at room temperature. After three wash steps with PBS, fluorescein-conjugated secondary pig anti-rabbit IgG (Dako, Glostrup, Denmark) was added at a 1:1000 dilution in PBS/BSA (5%) for 1 hr at room temperature. Actin stress fibres were stained using phalloidin-TRITC (Sigma-Aldrich) at a dilution of 1:1000 for 1 hr at room temperature. After immunohistochemical labelling, Vectashield mounting medium (Vector Laboratories, Burlingame, CA, USA) was used for mounting slides. Slides were analysed under an Axio Imager fluorescence microscope (Carl Zeiss AG, Oberkochen, Germany). Corresponding negative controls to estimate unspecific binding of secondary antibodies were handled similarly, but incubated in PBS/BSA without primary antibody.

### Cell viability assay

To analyse the CTGF effect on the viability of HTM-N and pSiCTGF-HTM-N cells after treatment with H_2_O_2_, cells were seeded into 96-well plates at 3.0 × 10^4^ cells/well. The cells were treated with 50 μM of H_2_O_2_ alone or in combination with 50 ng/ml of recombinant CTGF (rCTGF) 21 for 24 hrs under serum-free conditions. Viability of cells was analysed by a MTT (3-(4,5-dimethylthiazol-2-yl)-2,5-diphenyltetrazolium bromide) assay. The yellow MTT is reduced by a mitochondrial reductase to purple formazan in the mitochondria of living cells. The absorbance of the formed fomazan is measured by 570 nm wave length.The cells were washed with PBS, and 80 μl/well MTT solution (tetrazolium dye, PBS and serum-free medium) was added for 2 hrs. The formazan crystals that formed were dissolved by the addition of dimethyl sulfoxide (DMSO). Absorption was measured with a spectrophotometer (Sunrise, Tecan, Maennedorf, Switzerland) at 570 nm and analysed using Magellan software (Tecan). Results are expressed as the mean percentage of the controls. Untreated HTM-N and pSiCTGF-HTM-N cells served as controls. Values of each sample were normalized to a ‘blank’ containing DMSO only.

### Number of experiments and statistical analysis

To assess the effects of the different treatments, each experiment was repeated at least three times from primary HTM cell lines of three different donors and with the HTM-N and pSiCTGF-HTM-N cells. Student's *t*-test was used for statistical analysis and differences with a *P*-value smaller 0.05 were regarded as significant, and with a *P* < 0.01 as highly significant.

## Results

### Heat shock induces CTGF expression

**Figure 1:**
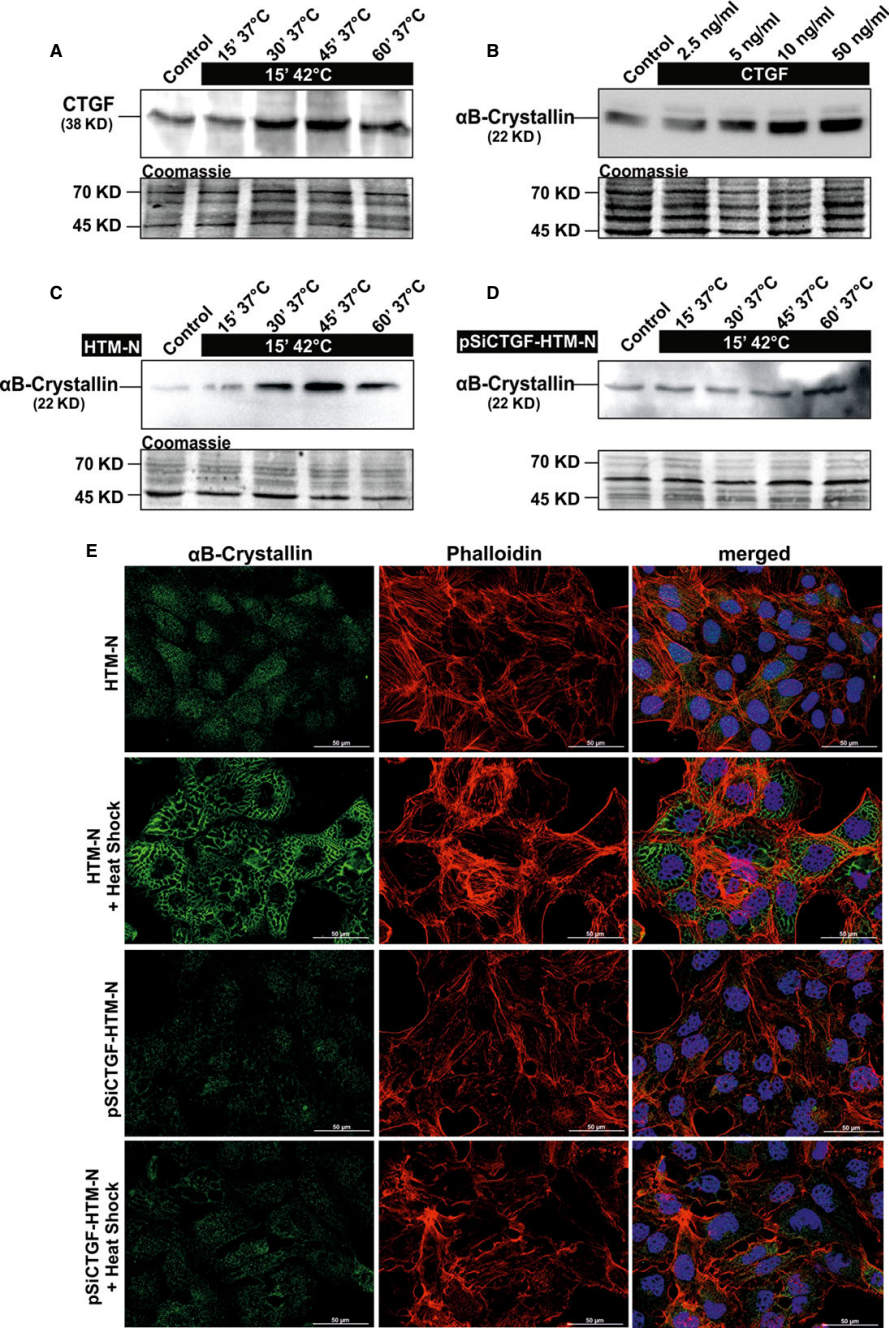
Effect of heat shock on the expression of CTGF and αB-crystallin. (A) Western blot analysis for CTGF in cell extract of HTM-N cells after heat shock at 42°C and recovery phase of 15–60 min. After 30 min. the CTGF expression was increased up to 3.3 ± 0.4-fold (*P* < 0.05) and maintained at this level until 60 min. (B) Western blot analysis for αB-crystallin in cell extract of HTM cells after treatment with 2.5–50 ng/ml of CTGF for 24 hrs. αB-crystallin was significantly increased after CTGF treatment for 24 hrs (3.2 ± 0.3-fold, *P* < 0.05). (C and D) Western blot analysis for αB-crystallin in cell extracts of HTM-N (C) and pSiCTGF HTM-N cells (D) after heat shock at 42°C and recovery phases of 15–60 min. Densitometric evaluation of HTM-N cells showed a maximum of 4.1 ± 0.3-fold after 30 min. (*P* < 0.05) while there was only a slight induction of 2.1 ± 0.5-fold observed after 60 min. in pSiCTGF HTM-N cells. Membranes were stained with coomassie blue to confirm equal loading of proteins. (E) Immunohistochemical staining of αB-crystallin (green) and actin fibres (phalloidin, red) in HTM-N and pSiCTGF HTM-N cells 30 min. after heat shock. HTM-N cells showed a more intense staining for αB-crystallin and an increased formation of actin stress fibres after heat shock, while there was no increase in pSiCTGF HTM-N cells. Scale bar = 50 μm.

As mechanical stress induces CTGF in HTM cells 31, we investigated whether CTGF is also induced by other stressors. To this end, HTM-N cells were treated with heat shock (42°C) and were then allowed to recover at 37°C. After 30 min., the expression of CTGF was significantly increased (3.3 ± 0.4-fold, *P* < 0.05) and remained at this level during the entire 60 min. of the recovery phase (Fig.1A).

### CTGF induces αB-crystallin expression

Amounts of the small heat-shock protein (sHSP) αB-crystallin are higher in the TM of POAG eyes 34 and are induced by TGF-β in cultured HTM cells 35. Here, we were interested to learn whether there is a link between αB-crystallin and CTGF in HTM cells. HTM cells treated with recombinant CTGF showed a dose-dependent increase in the amounts of αB-crystallin, which were 3.2 ± 0.3-fold higher after treatment with 10 and 50 ng/ml of CTGF for 24 hrs (*P* < 0.05, Fig.1B).

We now wanted to know whether the expression of αB-crystallin is changed in the presence of lower amounts of CTGF. For that purpose, we used the pSiCTGF-HTM-N cell line and compared it to HTM-N cells 11. HTM-N cells treated with 42°C for 15 min. showed an increased immunohistochemical staining for αB-crystallin and an increase in actin stress fibre formation after 30 min. of recovery phase (Fig.1E). Following Western blot experiments, densitometric analysis of the immunoblots identified a significant up-regulation of αB-crystallin in HTM-N cells (4.1 ± 0.3-fold, *P* < 0.05, 45 min., Fig.1D). In contrast in pSiCTGF-HTM-N cells, αB-crystallin staining and stress fibre formation was unchanged after heat shock exposure (Fig.1E). Western blot analysis of αB-crystallin synthesis in pSiCTGF-HTM-N cells showed a delayed and reduced induction in comparison to HTM-N cells. An induction (2.1 ± 0.5-fold) of αB-crystallin was only observed after 60 min. of recovery phase (Fig.1D).

### Oxidative stress induces CTGF expression

**Figure 2:**
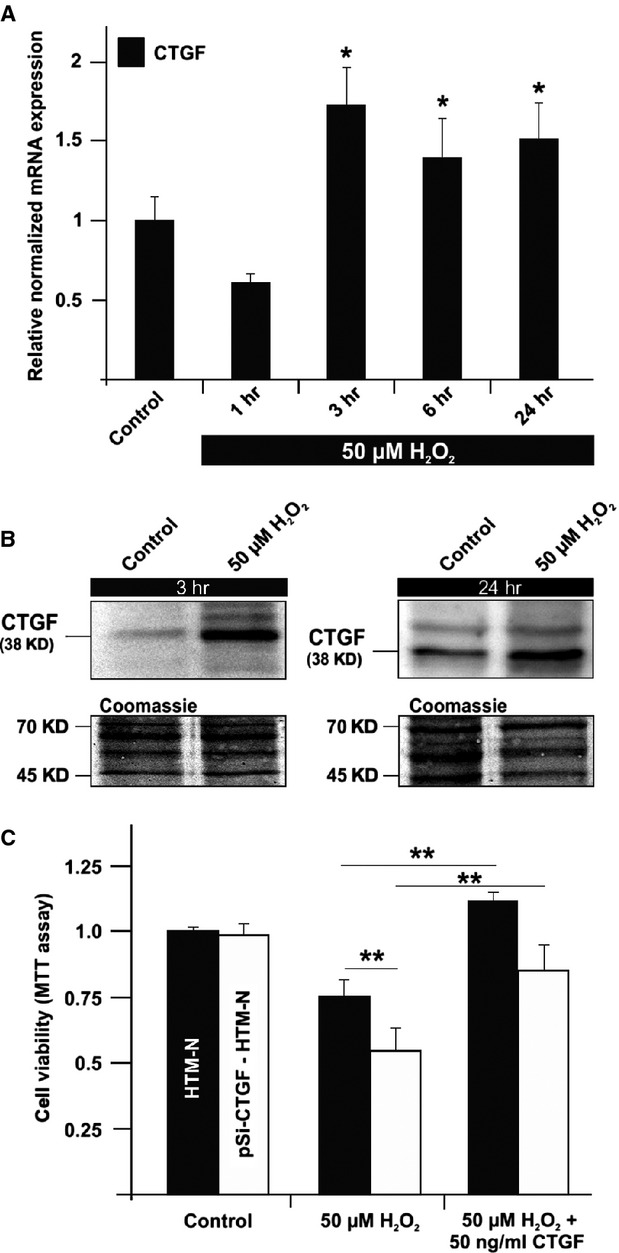
(A) Real-time RT-PCR analysis of CTGF mRNA expression in HTM cells after treatment with 50 μM H_2_O_2_ for 1–24 hrs. A 3-hrs treatment resulted in a significant increase in CTGF mRNA expression up to 1.8 ± 0.2-fold which remained up-regulated for at least 24 hrs. The mean value obtained from untreated cells was set at 1. GNB2L and GAPDH were used as reference genes. Means ± SD are shown. (B) Western blot analysis for CTGF in cell extract of HTM cells after treatment with 50 μM H_2_O_2_ for 3 and 24 hrs. Densitometric evaluation showed a maximum of 2.2 ± 0.3-fold after oxidative stress for 3 hrs. Membranes were stained with coomassie blue to confirm equal loading of proteins. (C) Measurement of cell viability in HTM-N and pSiCTGF HTM-N cells *via* MTT assay after treatment with 50 μM H_2_O_2_ alone or in combination with 50 ng/ml of CTGF for 24 hrs. Treatment of HTM-N cells lead to a significant reduction to 75% ± 5%. The decrease was more intense in pSiCTGF-HTM-N cells (55% ± 2%). Cells treated with a combination of H_2_O_2_ and CTGF showed a significant higher viability (in HTM-N 103% ± 2% and pSiCTGF-HTM-N 84% ± 1%). The mean value obtained from untreated cells was set at 1. Means ± SD are shown. Asterisks mark statistically significant (*P < 0.05) and high significant differences (**P < 0.01).

As increasing age comes along with higher amounts of reactive oxygen species (ROS) in the eye 36, we investigated whether oxidative stress influences the expression of CTGF in HTM cells. To this end, cells were treated with 50 μM H_2_O_2_ for 1, 3, 6 or 24 hrs and CTGF and its mRNA were determined by real-time RT-PCR and Western blotting. A 3-hrs treatment resulted in a significant increase in CTGF mRNA expression up to 1.8 ± 0.2-fold (*P* < 0.05) which remained upregulated for at least 24 hrs (Fig.2A). Moreover, after 3 hrs of oxidative stress, the amounts of CTGF were 2.2 ± 0.3-fold higher than in control cells (*P* < 0.05). After 24 hrs, the amounts of CTGF were still 1.9 ± 0.2-fold higher than in controls (*P* < 0.05; Fig.2B).

### CTGF protects HTM cells against oxidative stress

As CTGF appeared to be a primary response gene to stress and as the amounts of CTGF correlate with sHSP expression, we investigated whether CTGF affects the viability of H_2_O_2_-treated HTM-N cells. For this purpose, we treated HTM-N and pSiCTGF-HTM-N cells with 50 μM H_2_O_2_ alone or in combination with 50 ng/ml of CTGF for 24 hrs. Measuring cell viability was conducted by a MTT assay. Treatment of HTM-N cells with H_2_O_2_ lead to a significant reduction in cell viability to 75% ± 5% compared to untreated control cells (*P* < 0.05). The decrease in viability was significantly higher in pSiCTGF-HTM-N cells (55% ± 2%; *P* < 0.01). Cells treated with a combination of H_2_O_2_ and CTGF showed a significantly higher viability (in HTM-N: 103% ± 2%, *P* < 0.01 and pSiCTGF-HTM-N: 84% ± 1%; *P* < 0.01) in comparison to the H_2_O_2_-treated cells (Fig.2C).

### Treatment with LatA causes a reduction in the amounts of CTGF and its mRNA

**Figure 3:**
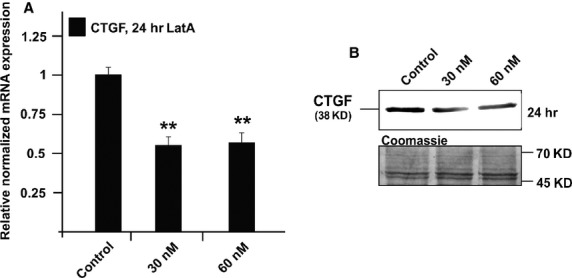
Analysis of CTGF expression in HTM cells after treatment with 30 or 60 nM latrunculin A for 24 hrs. (A) Real-time RT-PCR analysis of CTGF mRNA expression. The mean value obtained from untreated cells was set at 1. GNB2L and GAPDH were used as reference genes. Means ± SD are shown. Asterisk marks statistically significant differences between control and latrunculin A-treated cells (**P < 0.01). (B) Western blot analysis for CTGF in HTM cell extract. Densitometric evaluation showed a minimum of 0.67 ± 0.7-fold (*P < 0.01) compared to the untreated control after treatment with 60 nM for 24 hrs. Membranes were stained with coomassie blue to confirm equal loading of proteins. Asterisks mark statistically significant (*P < 0.05) and high significant differences (**P < 0.01).

Treatment of HTM cells with 30 nM LatA for 24 hrs led to a highly significant 0.55 ± 0.1-fold reduction in CTGF mRNA expression when compared to untreated control cells (Fig.3A; *P* < 0.01. A comparable effect was observed after treatment with 60 nM LatA, which did also cause a reduction in the amounts of CTGF (*P* < 0.01, Fig.3B).

### Treatment with ET-1, Ang II or IGF-1 induces CTGF and its mRNA

**Figure 4:**
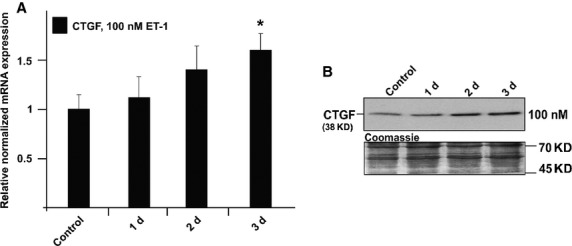
Analysis of CTGF expression in HTM cells after treatment with 100 nM endothelin-1 for different time periods. (A) Real-time RT-PCR analysis of CTGF mRNA expression. The mean value obtained from untreated cells was set at 1. GNB2L and GAPDH were used as reference genes. Means ± SD are shown. Asterisk marks statistically significant differences between control and endothelin-1-treated cells (*P < 0.05). (B) Western blot analysis for CTGF in HTM cell extract. Densitometric evaluation showed a maximum of 1.8 ± 0.2-fold compared to untreated control after 3 days. Membranes were stained with coomassie blue to confirm equal loading of proteins.

To assess the effects of ET-1 on the expression of CTGF in the TM, HTM cells were treated with ET-1 at a concentration of 100 nM for 1–24 hrs and 1 day to 3 days. The amounts of CTGF and its mRNA were analysed by real-time RT-PCR and Western blot analysis. Treatment for 1, 3 and 12 hrs did not cause significant changes in the expression of CTGF (data not shown). After 3 days of treatment, the expression of mRNA for CTGF increased significantly by 1.6 ± 0.2-fold (Fig.4A; *P* < 0.05). In addition, higher amounts of CTGF were detected in cell extracts of treated cells compared to untreated control cells (Fig.4B).

**Figure 5:**
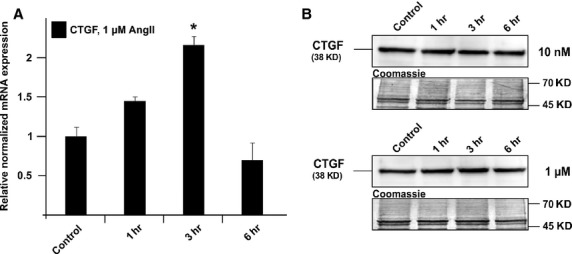
Analysis of CTGF expression in HTM cells after treatment with 10 nM or 1 μM angiotensin-II for different time periods. (A) Real-time RT-PCR analysis of CTGF mRNA expression. The mean value obtained from untreated cells was set at 1. GNB2L and GAPDH were used as reference genes. Means ± SD are shown. Asterisk marks statistically significant differences between control and angiotensin-II-treated cells (*P < 0.05). (B) Western blot analysis for CTGF in HTM 71 cell extract. Densitometric evaluation showed a maximum of 2.6 ± 0.8-fold compared to the untreated control after treatment with 1 μM for 3 hrs. Membranes were stained with coomassie blue to confirm equal loading of proteins.

To study the effects of Ang II, HTM cells were treated for 1, 3 or 6 hrs with concentrations of 10 nM and 1 μM of Ang II and analysed as described above. Following treatment with 10 nM Ang II, the amounts of CTGF and its mRNA remained unaffected (*P* > 0.05, Fig.5B). In contrast, treatment with 1 μM of Ang II led to significant changes after 3 hrs of treatment. The expression of mRNA for CTGF increased up to 2.16 ± 0.18-fold (Fig.5A; *P* < 0.05), an effect that correlated with the presence of higher amounts of CTGF (Fig.2B). When cells were treated for 6 hrs, no significant changes were observed when compared with control cells (Fig.5B).

**Figure 6:**
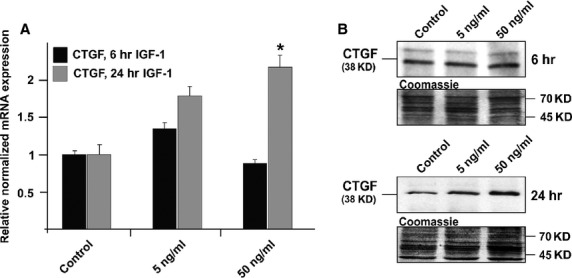
Analysis of CTGF expression in HTM cells after treatment with 5 or 50 ng/ml IGF-1 for different time periods. (A) Real-time RT-PCR analysis of CTGF mRNA expression. The mean value obtained from untreated cells was set at 1. GNB2L and GAPDH were used as reference genes. Means ± SD are shown. Asterisk marks statistically significant differences between control and IGF-1-treated cells (*P < 0.05). (B) Western blot analysis for CTGF in HTM cell extract. Densitometric evaluation showed a maximum of 2.1 ± 0.3-fold (*P < 0.05) compared to the untreated control after treatment with 50 ng/ml for 24 hrs. Membranes were stained with coomassie blue to confirm equal loading of proteins.

For studies on the CTGF-inducing effect of IGF-1, HTM cells were treated with IGF-1 at concentrations of 5 and 50 ng/ml for 6 and 24 hrs respectively. After 6 hrs of incubation, there was no significant increase between IGF-1-treated and control cells with respect to the amounts of CTGF and its mRNA. In contrast, treatment with 50 ng/ml of IGF-1 for 24 hrs led to a significant 2.2 ± 0.2-fold increase of CTGF mRNA when compared to untreated control cells (Fig.6A; *P* < 0.05). The increase in mRNA caused a significant increase in the amounts of CTGF (*P* < 0.05, 2.1 ± 0.3-fold, Fig.6B).

## Discussion

We conclude that CTGF is a primary response gene after exposure to stress that protect HTM cells from injury. The protective effect of CTGF appears to be associated with a stabilization of the actin cytoskeleton. This conclusion is based on (*i*) the findings that CTGF is immediately upregulated in HTM cells after treatment with different stressors and (*ii*) that CTGF induces the expression of the sHSP αB-crystallin. (*iii*) The conclusion is supported by the observation that a knockdown of CTGF leads to a reduced cell viability in HTM cells after exposure to stress and to an attenuated up-regulation of αB-crystallin, (*iv*) whereas a pretreatment with CTGF could prevent the stress-induced cell loss.

The cellularity of the TM decreases throughout life 37,38 and the loss of TM cells is more pronounced in patients with POAG 39. The causes for the loss of TM cells are not clear, but it is known that TM cells are exposed to many different stressors during the ageing process 40,41. Among the stressors is mechanical stress mediated by ciliary muscle contraction and aqueous flow rate changes. Mechanical stress is thought to be elevated in POAG patients with increasing IOP 40, a major risk factor for glaucoma. Besides mechanical stress, TM is constantly exposed to oxidative stress 41.

In many diseases, it is accepted that the ageing process is associated with a higher exposure rate to free radicals, which would lead to a decline of physiological functions of various tissues 42 and several studies suggest that an increased formation of ROS might be associated with glaucoma 36,43. In POAG patients, a significant reduction in mitochondrial respiratory activity and a significant decrease in anti-oxidative capacity were detected 41,44–46. Those findings resulted in the hypothesis that oxidative stress could be involved in onset and progression of POAG. Here, we show that CTGF is a primary response gene after oxidative stress in TM cells. The data are supported by *in vivo* analysis of early response genes after oxidative stress. In mice, the induction of oxidative stress in the cerebellum led to a substantial increase in CTGF within 6 hrs 47. The immediate up-regulation of CTGF under stress conditions was further confirmed by our heat-shock experiments. Together with the known findings that also mechanical stress is able to induce CTGF expression 31, we conclude that CTGF might be a general primary response gene to various kinds of stressors in HTM cells.

The physiological function of the early up-regulation of CTGF seems to be a protective mechanism in HTM cells. The supplementation of CTGF prior to H_2_O_2_ treatment had a beneficial effect on the viability of TM cells. A potential role for CTGF in cell survival was shown in gallbladder cancer cells, where silencing of CTGF led to a reduced cell viability 48. We could observe a similar effect in TM cells, where reduced levels of CTGF led to a decline in cell viability rate after oxidative stress, whereas adding CTGF partially rescued the loss of TM cells. A protective function of CTGF was previously shown in the kidney, where supplementation of CTGF guarded puromycin-treated podocytes from cell death 49.

The protective effect of CTGF might be directly linked to the expression of the sHSP αB-crystallin, as CTGF treatment led to a significant up-regulation of αB-crystallin in HTM cells. αB-crystallin belongs to the family of sHSPs, and it is known to be up-regulated in the TM of POAG patients 34. The increased presence of sHSPs might have a protective effect, as TM cells respond to oxidative stress and heat shock by αB-crystallin induction 50, whereas silencing of CTGF in TM cells blocked the stress-induced up-regulation of the αB-crystallin. As both proteins are simultaneously regulated during the exposure to heat shock, we assume that CTGF acts as modulator of the αB-crystallin synthesis, because of the matricellular character of CTGF 51. sHSPs are able to protect cells by different mechanisms depending on their subcellular localization. Under stress conditions, αB-crystallin can translocate to the mitochondria and thereby interacting with various components of the mitochondrial apoptotic machinery and preventing cell death 52,53, whereas the cytosolic αB-crystallin can inhibit actin depolymerization, thereby leading to an increased cell survival 54. We assume that CTGF protects the cells against the oxidative stress-induced disruption of the cytoskeleton and disaggregation of actin fibres, a critical point for cell survival 54. In an earlier study, we could already show the positive effect of CTGF on formation of actomyosin fibres and the contractility in HTM cells 11, whether the mitochondrial apoptotic events are also altered after CTGF treatment have to be investigated in the future.

Based on our observations, we wanted to address additionally the question whether CTGF regulation in HTM cells is also linked to other factors, which are present in the AH and/or are involved in the outflow facility regulation and are assumed to be involved in CTGF regulation in other tissues.

In the context of a CTGF-mediated induction of ECM synthesis, we also investigated the effect of IGF-1 on CTGF expression. IGF-1 stimulates CTGF to induce collagens *via* binding to the IGF-binding domain of CTGF 55. IGF-1 is present in the AH 56 and is expressed in the TM together with its receptors 57. In studies on the signalling pathways of IGF-1, the RhoA/ROCK signalling pathway is among the most commonly highlighted 58. In our study, physiological concentrations of IGF-1 led to an increased expression of CTGF in TM cells 56. Little is known though about the function of IGF-1 and its receptor within the TM, an avenue that should be analysed in the future.

Clearly, the common pathway between the molecules investigated here appears to be the RhoA/ROCK pathway that is involved in the regulation of outflow facility by altering the actin cytoskeleton of the TM. Previous studies showed that Ang II and ET-1 induce CTGF expression *via* the small GTPase RhoA 59,60. In this study, we obeserved a late induction of CTGF after ET-1 treatment indicating that the increase in CTGF could be a secondary effect. On the other hand, Horstmeyer *et al*. observed a pronounced late induction of CTGF after ET-1 in comparison to short time treatments. This finding was explained with different expression patterns of the two endothelin receptors A and B at the different points 61. In contrast, a rapid increase in CTGF after Ang II treatment clearly implicates a direct effect of the substance. The fast decline of the CTGF expression under the basal levels might be because of the short half-life of Ang II.

The CTGF-inducing effect of RhoA was shown by the treatment of HTM cells with a constitutive active form of RhoA, whereas the inhibition of the Rho kinase, a downstream mediator of RhoA, lead to a substantial down-regulation of CTGF 62. The CTGF expression is coupled to the stability of the actin cytoskeleton 63, the formation of F-actin stress fibres led to an increased CTGF expression. This regulation was dependent on the availability of G-actin. Actin disrupting agents like LatA and B, which cause an enhanced cellular content of G-actin, lead to a reduction in CTGF expression in different cell lines 64. Quite similarly, the LatA treatment of HTM cells caused a significant down-regulation of CTGF. As latrunculin treatment of perfused anterior chambers lead to an increase in outflow facility 65,66, we speculate that, given the loss of contractility in TM cells, the decrease in CTGF-mediated ECM deposition might contribute in the long run to the outflow increasing effect of latrunculins. On the other hand, under stress, like increased mechanical load or oxidative stress, the TM cells react with increased F-actin stress fibres formation, leading to decreased G-actin levels and thereby to an enhanced CTGF expression. Thereby, inducing a vicious circle, as CTGF by itself can activate the RhoA/ROCK signalling pathway 11. If the stress last only for a short period, the expression of CTGF declines rapidly to a basal expression, which led to the assumption that compensatory mechanisms are activated to normalize the CTGF expression levels 67. Under pathological conditions, which were shown in other tissues, those mechanisms seem to fail and the CTGF up-regulation maintains in those tissues leading to fibrotic changes 68,69.

We conclude that CTGF is an important regulatory molecule in the TM and that the RhoA/ROCK signalling pathway is involved in some CTGF-mediated TM effects 11,62,70. The newly identified molecules and stressors, together with the findings that CTGF is induced by dexamethasone, TGF-β1 and 2 in HTM cells 31,32, brings up the idea that CTGF may play an important role in the pathogenesis of glaucoma. Along this line, the reported constitutive basal expression of CTGF in the TM 25 could be necessary for maintenance of the TM actin cytoskeleton and ECM. An immediate upregulation of CTGF following short-term stress might be an important mechanism to protect TM cells from damage and death. In contrast, a continuous upregulation of CTGF after chronic cellular stress might significantly alter TM homoeostasis and lead to an increase in TM ECM, actin-mediated contractility and finally stiffness. Clearly, such a scenario is likely to result in increased outflow resistance and IOP, and finally POAG.
